# Shorter telomere lengths in patients with severe COVID-19 disease

**DOI:** 10.18632/aging.202463

**Published:** 2021-01-11

**Authors:** Raul Sanchez-Vazquez, Ana Guío-Carrión, Antonio Zapatero-Gaviria, Paula Martínez, Maria A. Blasco

**Affiliations:** 1 Telomeres and Telomerase Group, Molecular Oncology Program, Spanish National Cancer Centre (CNIO), Melchor Fernández Almagro 3, Madrid, Spain; 2 Field Hospital COVID-19, IFEMA, Madrid, Spain

**Keywords:** SARS-CoV-2, COVID-19, telomeres, aging

## Abstract

The incidence of severe manifestations of COVID-19 increases with age with older patients showing the highest mortality, suggesting that molecular pathways underlying aging contribute to the severity of COVID-19. One mechanism of aging is the progressive shortening of telomeres, which are protective structures at chromosome ends. Critically short telomeres impair the regenerative capacity of tissues and trigger loss of tissue homeostasis and disease. The SARS-CoV-2 virus infects many different cell types, forcing cell turn-over and regeneration to maintain tissue homeostasis. We hypothesize that presence of short telomeres in older patients limits the tissue response to SARS-CoV-2 infection. We measure telomere length in peripheral blood lymphocytes COVID-19 patients with ages between 29 and 85 years-old. We find that shorter telomeres are associated to increased severity of the disease. Individuals within the lower percentiles of telomere length and higher percentiles of short telomeres have higher risk of developing severe COVID-19 pathologies.

## INTRODUCTION

The current COVID-19 pandemic (https://www.who.int/) is produced by the SARS-CoV-2 virus, a novel zoonotic Coronavirus of the betacoronavirus genus that most likely crossed species from bats to humans leading to a pneumonia outbreak initially reported in Wuhan, China and now affecting the majority of countries. SARS-CoV-2 causes from mild flu-like symptoms in approximately 80% of the cases to a severe lung and multi-organic failure which can result in death of a significant percentage of patients. Pathologies associated with SARS-CoV-2 include severe lung failure, diarrhea, heart infarct, and brain pathologies among others [1–3]. This wide viral tropism is mediated by expression of the Angiotensin-converting enzyme 2 (ACE2), which acts as the receptor protein for the virus to enter the host cells. In particular, the SARS-CoV-2 spike protein directly binds the ACE2 human protein [4–7]. The human ACE protein is expressed in alveolar type II (ATII) cells in the lung [8], as well as in the kidney, the heart and the gut [9–14]. This expression pattern of the ACE protein explains that a preferential site for SARS-CoV-2 infection is the lung [4, 15, 16], although the virus can also infect kidney, intestine, and heart cells causing severe pathologies in all these tissues [1–3, 11, 17, 18]. In this regard, it caught our attention that a common outcome of SARS-CoV-2 infection seems to be induction of fibrosis-like phenotypes in the lung and kidney, suggesting that the viral infection maybe exhausting the regenerative potential of tissues [11, 16–18].

In contrast to influenza infection, that causes a high mortality in infants [19–24], SARS-CoV-2 infection causes low mortality in infants or children but results in a progressively increased mortality with increasing age reaching up to 15% mortality in individuals that are ≥80 years old (see https://covid19.isciii.es/ for mortality data in Spain). These findings suggest that molecular mechanisms at the origin of organismal aging maybe influencing the outcome of SARS-CoV-2 infection by increasing lethality. One of such molecular events underlying aging is the progressive shortening of telomeres throughout life, which can cause exhaustion of the proliferative potential of stem cells and immune cells among others [25–27].

Telomeres are specialized structures at the chromosome ends, which are essential for chromosome-end protection and genomic stability [28]. Vertebrate telomeres consist of tandem repeats of the TTAGGG DNA sequence bound by a six-protein complex known as shelterin, which prevents chromosome end-to-end fusions and telomere fragility [29, 30]. As cells divide and DNA has to be replicated, telomeres become progressively shorter owing to the so-called “end replication problem” [31, 32]. Thus, telomere shortening occurs associated with increasing age in humans [33], mice [34] and other species, and the rate of telomere shortening has been shown to correlate with species lifespan [35]. When telomeres become critically short this results in loss of telomere protection, leading to activation of a persistent DNA damage response [36] and loss of cellular viability by induction of apoptosis and/or senescence [30].

Telomerase is a reverse transcriptase that is able to elongate telomeres *de novo* by adding TTAGGG repeats to chromosome ends [37]. Telomerase is active in embryonic stem cells, thereby ensuring sufficiently long telomeres with generations in a given species. After birth, however, telomerase expression is silenced in the majority of cell types causing telomeres to shorten with age.

We have shown by using telomerase-deficient mice with critically short telomeres, that short telomeres are sufficient to impair the ability of stem cells to regenerate different tissues, including skin, brain and bone marrow [38–41]. In humans, mutations in telomerase or telomere-binding proteins can also lead to very short telomeres and appearance of pathologies characterized by loss of the regenerative capacity of tissues and presence of fibrosis in lungs, liver or kidney, as well as by intestinal atrophy and bone marrow aplasia [42].

In particular, we previously demonstrated that short or dysfunctional telomeres are at the origin of pulmonary fibrosis in mouse models of the disease [43]. In particular, induction of telomere dysfunction specifically in alveolar type II (ATII) cells by deletion of an essential telomere protective protein in these cells, TRF1, is sufficient to induce progressive and lethal pulmonary fibrosis phenotypes in mice, which are concomitant with induction of telomeric DNA damage, cell death and senescence [43]. These findings demonstrate that dysfunctional telomeres in lungs ATII cells lead to loss of viability of these cells and induction of fibrosis. Also in support of this notion, we have demonstrated that therapies aimed to elongate telomeres, such as a telomerase gene therapy using adeno-associated vectors (AAV9-TERT) can stop the progression of pulmonary fibrosis associated to short telomeres in mouse models of the disease by increasing telomere length in ATII cells, as well as their proliferative potential [44], thus demonstrating the importance of sufficiently long telomeres to allow tissue regeneration.

Importantly, as SARS-CoV-2 infects different cell types in humans, including ATII cells in the lungs, it is plausible that viral infection could damage these different cell types forcing an increased turn-over of different regenerative cell types. While in young individuals with sufficiently long telomeres, regenerative cell types, such as lung ATII cells could undergo these extra cell divisions and contribute to tissue healing, older individuals with shorter telomeres may fail to allow cell proliferation and regeneration, thus leading to tissue failure. Thus, here we set to assess whether telomere length in COVID-19 patients correlated with development of more severe COVID-19 pathologies.

## RESULTS

### Pathologies in COVID-19 patient cohort

In order to assess the potential impact of telomere length on pathologies associated to COVID-19 disease, we obtained both DNA and mononuclear cells from peripheral blood samples from patients hospitalized at the IFEMA field hospital in Madrid, which was constructed to treat COVID-19 patients. A total of 61 female and 28 male patients of ages ranging from 29 to 85 years old were included in the study (Table 1). The patient cohort had different severity of pathologies and received the treatments indicated in Table 1. None of the patients included in this study died as a consequence of the COVID-19 disease.

**Table 1:** Patients included in this study.

**Age**	**Sex**	**COVID-19 severity**	**Treatment**
29	Male	Moderate	Hydroxychloroquine, Azitromicine
30	Female	Moderate	Dolquine, Azitromicine
31	Female	Moderate	Hydroxychloroquine, Azitromicine, Kaletra
33	Female	Moderate	Hydroxychloroquine, Azitromicine
33	Female	Severe	Hydroxychloroquine, Azitromicine
35	Male	Moderate	Hydroxychloroquine, Ceftriaxone, Azitromicine, Kaletra
36	Female	Acute	Hydroxychloroquine, Kaletra, Corticoids, Ceftriaxone, Azitromicine, LINEZOLID
38	Female	Mild	Hydroxychloroquine
39	Female	Moderate	Hydroxychloroquine, Azitromicine
40	Male	Severe	Hydroxychloroquine, Azitromicine, Ceftriaxone, systemic corticoids
41	Female	Moderate	Hydroxychloroquine, Kaletra
42	Male	Severe	Hydroxychloroquine, Kaletra, Corticoids
43	Female	Mild	Hydroxychloroquine, Azitromicine
43	Female	Severe	Hydroxychloroquine, Azitromicine, Corticoids, Kaletra, Tocilizumab
43	Female	Moderate	Hydroxychloroquine, Azitromicine
44	Male	Mild	Hydroxychloroquine
45	Female	Moderate	Dolquine, Kaletra, Azitromicine
45	Female	Severe	Kaletra, Hydroxychloroquine, Azitromicine, Corticoids
46	Female	Moderate	Kaletra, Dolquine, Colchicine
46	Female	Severe	Dolquine, Kaletra, Azitromicine, Corticoids
46	Female	Severe	Hydroxychloroquine, Azitromicine
47	Female	Moderate	Hydroxychloroquine
47	Male	Severe	Hydroxychloroquine, Azitromicine, systemic corticoids
47	Female	Mild	Hydroxychloroquine, Ceftriaxone
48	Male	Severe	Hydroxychloroquine, Azitromicine, Tocilizumab, Kaletra
49	Female	Moderate	Hydroxychloroquine, Azitromicine
49	Male	Severe	Hydroxychloroquine, Azitromicine, systemic corticoids, Tocilizumab
49	Male	Moderate	Hydroxychloroquine, Azitromicine
49	Male	Moderate	Hydroxychloroquine, Azitromicine, Ceftriaxone
49	Male	Moderate	Hydroxychloroquine, Kaletra
49	Female	Moderate	Hydroxychloroquine, Azitromicine
50	Female	Moderate	Hydroxychloroquine, Azitromicine
51	Male	Severe	Hydroxychloroquine, Azitromicine
51	Female	Moderate	Hydroxychloroquine, Azitromicine, Kaletra, Ceftriaxone
51	Female	Moderate	Hydroxychloroquine, Kaletra, Azitromicine, Corticoids
52	Female	Severe	Chloroquine, Kaletra, Tocilizumab, methylprednisolone
52	Male	Severe	Hydroxychloroquine
52	Female	Moderate	Hydroxychloroquine, Azitromicine
53	Female	Severe	Hydroxychloroquine, Corticoids, Tocilizumab
53	Male	Moderate	Hydroxychloroquine, Azitromicine
53	Male	Severe	Hydroxychloroquine, Azitromicine, Cortocoid therapy
54	Female	Acute	Hydroxychloroquine, Kaletra, Azitromicine, INTERFERON, Tocilizumab, Corticoids
54	Female	Acute	Hydroxychloroquine, Azitromicine, systemic corticoids, Tocilizumab
54	Female	Severe	Hydroxychloroquine, Azitromicine, Kaletra, systemic corticoids
54	Female	Severe	Hydroxychloroquine, Azitromicine, Corticoids, Tocilizumab
54	Female	Moderate	Hydroxychloroquine
54	Female	Moderate	Hydroxychloroquine, Azitromicine, Ceftriaxone
54	Female	Moderate	Hydroxychloroquine, Kaletra, Azitromicine
55	Female	Mild	Hydroxychloroquine
55	Female	Mild	Ceftriaxone, Hydroxychloroquine
55	Male	Moderate	Hydroxychloroquine
56	Female	Moderate	Dolquine, Azitromicine
56	Female	Severe	Hydroxychloroquine, Azitromicine, systemic corticoids
56	Male	Moderate	Hydroxychloroquine, Azitromicine
56	Female	Severe	REMDESIVIR, Corticoids, Tocilizumab
57	Male	Severe	Chloroquine, Corticoids, Interferon beta
57	Male	Acute	Hydroxychloroquine, Azitromicine, Corticoids, Tocilizumab
57	Female	Severe	Hydroxychloroquine, Kaletra, Corticoids
57	Male	Moderate	Hydroxychloroquine, Kaletra
57	Female	Severe	Hydroxychloroquine, Corticoids, Tocilizumab, Azitromicine
58	Male	Severe	Hydroxychloroquine, Azitromicine, systemic corticoids
58	Female	Moderate	Hydroxychloroquine+Azitromicine
58	Female	Severe	Hydroxychloroquine, Azitromicine, Methylprednisone
59	Female	Severe	Hydroxychloroquine, Azitromicine, Corticoids
59	Female	Moderate	Hydroxychloroquine, Kaletra, Predisolone
60	Female	Severe	Hydroxychloroquine, Azitromicine, Corticoids, Tocilizumab
60	Female	Moderate	Hydroxychloroquine+Azitromicine+Kaletra
61	Female	Moderate	Dolquine, Azitromicine
61	Male	Severe	Hydroxychloroquine, Ceftriaxone, Azitromicine, systemic corticoids
62	Female	Moderate	Hydroxychloroquine, Azitromicine
62	Female	Severe	Hydroxychloroquine, Azitromicine, Kaletra
63	Female	Severe	Hydroxychloroquine, Azitromicine, Dexamethasone
63	Male	Severe	Hydroxychloroquine, Azitromicine, Corticoids
65	Male	Severe	Hydroxychloroquine, Kaletra, Corticoids
65	Male	Severe	Hydroxychloroquine, Azitromicine, Kaletra, Dexamethasone, Tocilizumab
66	Female	Severe	Hydroxychloroquine, Kaletra, systemic corticoids
67	Female	Moderate	Hydroxychloroquine
67	Female	Severe	Chloroquine, Kaletra, Azitromicine, Tocilizumab, Corticoids
67	Male	Severe	Hydroxychloroquine, Azitromicine, Corticoids, Kaletra
69	Male		
70	Female	Severe	Dolquine, Kaletra, Azitromicine, Corticoids
71	Male	Severe	Hydroxychloroquine, Kaletra, Corticoids
71	Female	Severe	Hydroxychloroquine, Azitromicine, Cortocoid therapy
72	Female	Acute	Hydroxychloroquine, Azitromicine, systemic corticoids
73	Female	Severe	Hydroxychloroquine, Azitromicine, Kaletra, systemic corticoids, Tocilizumab
74	Female	Severe	Hydroxychloroquine, Azitromicine, Corticoids
77	Female	Severe	Hydroxychloroquine
81	Female	Mild	Hydroxychloroquine, Azitromicine, Ceftriaxone
85	Female	Moderate	Hydroxychloroquine, Kaletra, Azitromicine

In order to correlate patient severity with telomere length, we first grouped the patients according the a severity score ranging from 1 to 4, with severity score 1 in the case of patients with low fever and cough but without any radiological features of pneumonia to patients with severity score of 4 in the cases of patients with features of Acute Respiratory Distress Syndrome (ARDS) requiring mechanical ventilation along with presence of multiorgan dysfunction failure, metabolic acidosis and coagulation dysfunction (Materials and Methods).

### Determination of telomere length in COVID-19 patients

In order to determine telomere length in our patient cohort, peripheral blood was extracted from the arm from the different patients and used to measure telomere length by three independent techniques (Materials and Methods). First, we determined telomere length in DNA extracted from peripheral blood by using both the Southern blotting-based Telomere Restriction Analysis (TRF; see [38]) and the quantitative-PCR (qPCR) telomere length analyses [45]. In addition to these two technologies based on DNA samples, we also measured telomere length on fresh peripheral blood mononuclear cells by using the more precise high-throughput quantitative fluorescence *in situ* hybridization (HT Q-FISH) previously described by us [34, 35, 46], which allows determination of individual telomere fluorescence signals using tens of thousands of cells from a single patient. The fact that HT Q-FISH can determine individual telomere fluorescence spots in interphasic nuclei, each spot usually formed by several clustered telomeres, allows to the determine the abundance of very short telomeres.

**Figure 1:**
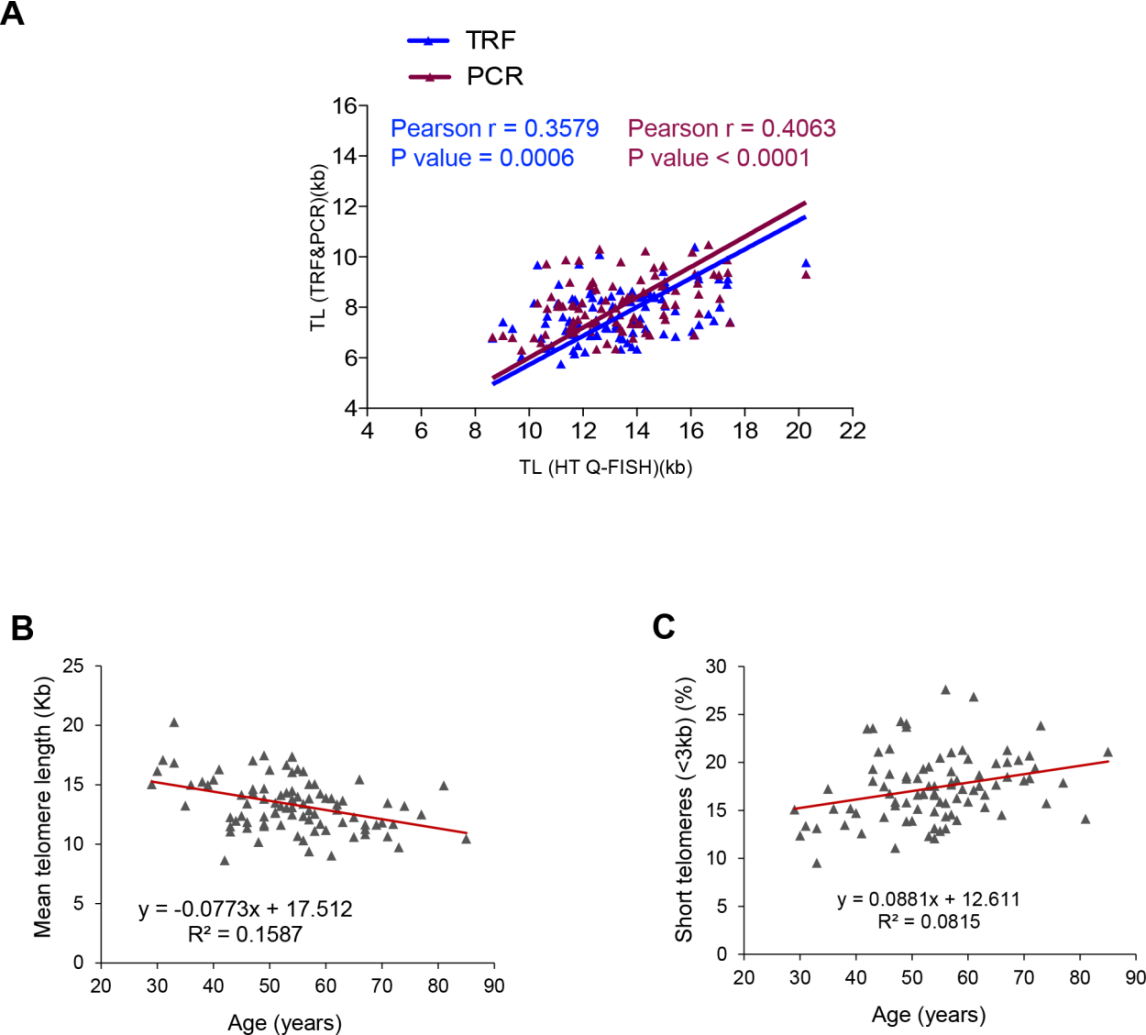
**Correlation between HT Q-FISH and PCR and TRF techniques for telomere length measurements.** (**A**) Correlation of telomere length measured by TRF, qPCR and HT Q-FISH in Peripheral blood mononuclear cell (PMBC) samples from 89 individuals. (**B**, **C**) Linear regression analysis was used to determine the rate of telomere shortening (**B**) and the rate of the increase of short telomeres (<3kb) (**C**) per year in PMBCs. The telomere length data in (**B**, **C**) correspond to HT-qFish analysis. The Pearson correlation coefficient and linear regression equation were determined using GraphPad Software.

We observed a very significant correlation between the telomere length measurements obtained by the three different techniques (Figure 1A), thus indicating the robustness of the data on telomere length obtained in our patient cohort. Given the good correlation of the telomere length data obtained with the three technologies, we decided to perform the rest of the analysis with the telomere length data obtained by HT Q-FISH, as it measures telomeres in a single cell manner and it also allows to measure individual telomere spots within single nuclei.

The rate of telomere shortening in the patient cohort was of 77 bp/year (Figure 1B). This rate of telomere shortening is in the range previously published by us and others [45–48]. In agreement with telomere shortening with increasing patient age, we also observed an increase in the abundance of short telomeres (ie, telomere fluorescence spots corresponding to less than 3 kb of telomere length) which increased at a rate of 8.8 % per year (Figure 1B).

**Figure 2:**
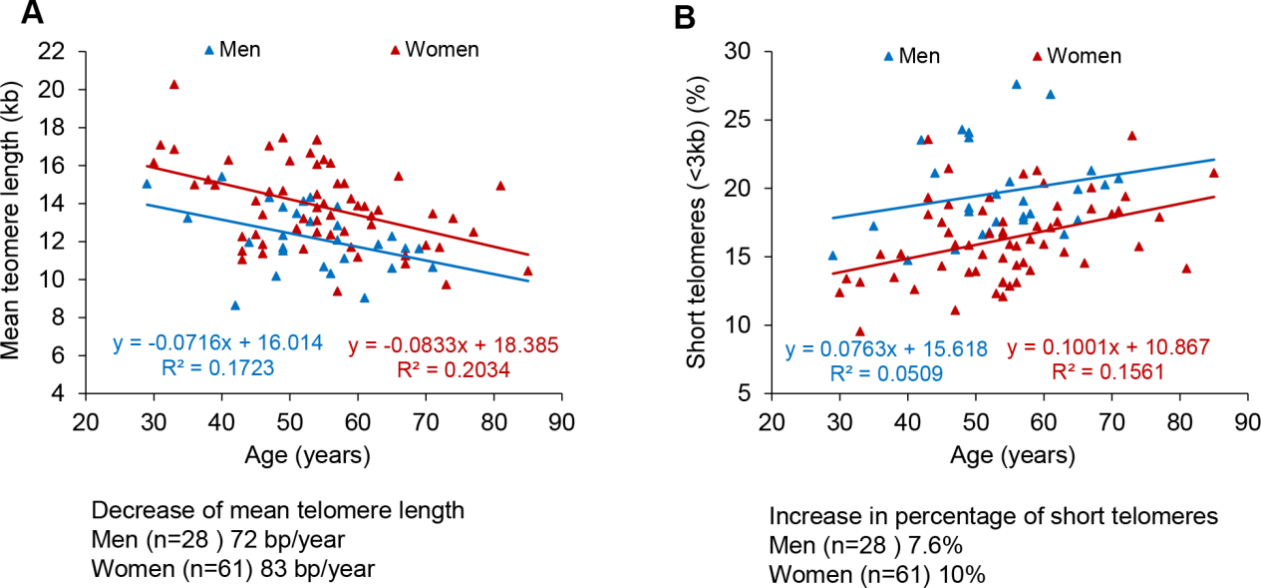
**Telomere shortening and of increase in short telomeres with age in men and women.** (**A**, **B**) Percentage of short telomeres (<3 kb) in PMBC samples. Mean telomere length (**A**) and percent of short telomeres (<3kb) (**B**) in PMBCs from male (blue) and female (red) patients. Linear regression analysis was used to assess the rate of telomere shortening expressed as number of bp loss and the increase of the percentage of short telomeres per year.

When we analyzed the data segregated by gender, linear regression of telomere length data in COVID-19 female patients showed that their telomeres were consistently longer than those of male patient at all age ranges, as well as they showed a lower percentage of short telomeres compared to male patients (Figure 2A–2D), also in agreement with previous findings [46]. Again, when segregated by gender, the rates of telomere shortening were in a range of 70-80 bp/year (Figure 2A). Similarly, the increase in the percentage of short telomeres with age was also similar in both genders (Figure 2A, 2B).

The fact that COVID-19 female patients had longer telomeres than men patients at different age ranges is in line with the fact that female COVID-19 patients show a lower mortality than males (see COVID-19 Sex-Disaggregated Data Tracker available at: http://globalhealth5050.org/covid19).

### Age and telomere length correlate with COVID-19 severity

In order to assess whether short telomeres correlated with the severity of COVID-19 disease, we used a Pearson correlation analysis between the mean telomere length or the percentage of short telomeres (<3 kb) as determined by the HT Q-FISH technique, and either age or the severity score of the different COVID-19 patients ranging from 1 (less severe) to 4 (more severe) (see Materials and Methods).

**Figure 3:**
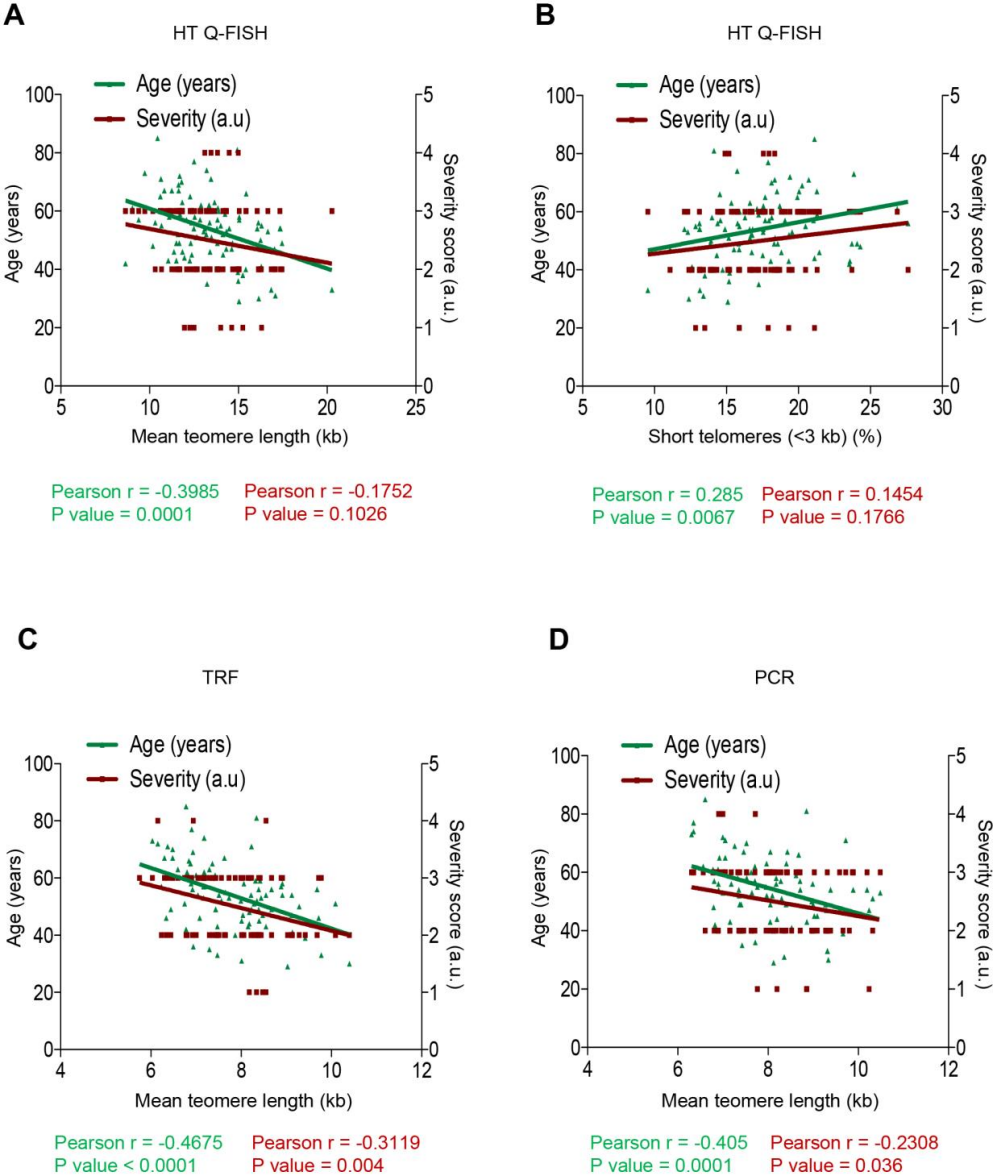
**Correlation between telomere length, age and COVID-19 severity.** (**A**–**D**) Pearson correlation analysis between telomere length (**A**, **C**, **D**) or percentage of short telomeres (<3 kb) (**B**) and age or COVID-19 severity in PMBC samples. In (**A**, **B**) telomere length was analysed by HT-QFISH and in (**C**, **D**) by TRF and PCR, respectively. The severity score was established by assigning values of 1, 2, 3, 4 for mild, moderate, severe, and acute, respectively (see Materials and Methods). The Pearson r coefficient and the P values are indicated.

As expected, we observed a significant inverse correlation between mean telomere length (TL) and age of the COVID-19 patients (r=-0.3985; p=0.0001; Figure 3A). We also observed a significant direct correlation between percentage of short telomeres (ie, telomeres < 3Kb) and patient age (r=0.285; p=0.0067; Figure 3B). Thus, these findings confirm a significantly higher incidence of short telomeres with increasing age in the COVID-19 patients. We also observed an inverse correlation between mean telomere length (TL) and the severity score of the COVID-19 patients when using HT Q-FISH (r=-0.1752; p=0.1026; Figure 3A) and a direct correlation between the percentage of short telomeres (ie, telomeres < 3Kb) and the severity score (r=0.1454; p=0.1766; Figure 3B), although these correlations did not reach statistical significance. To further analyze this, we performed similar analysis with the telomere length data obtained by TRF and by PCR (Figure 3C, 3D). Again, we confirmed a significant inverse correlation between mean telomere length (TL) and age of the COVID-19 patients by TRF (r=-0.4675; p<0.0001; Figure 3C) as well as by PCR (r=-0.405; p=0.0001; Figure 3D) techniques. Importantly, by these two DNA-based techniques, the correlation between telomere length and COVID-19 severity reached statistical significance (TRF: r=-0.3119, p=0.004; Figure 3C; PCR: r= -0.2308, p=0.036; Figure 3D.

**Figure 4:**
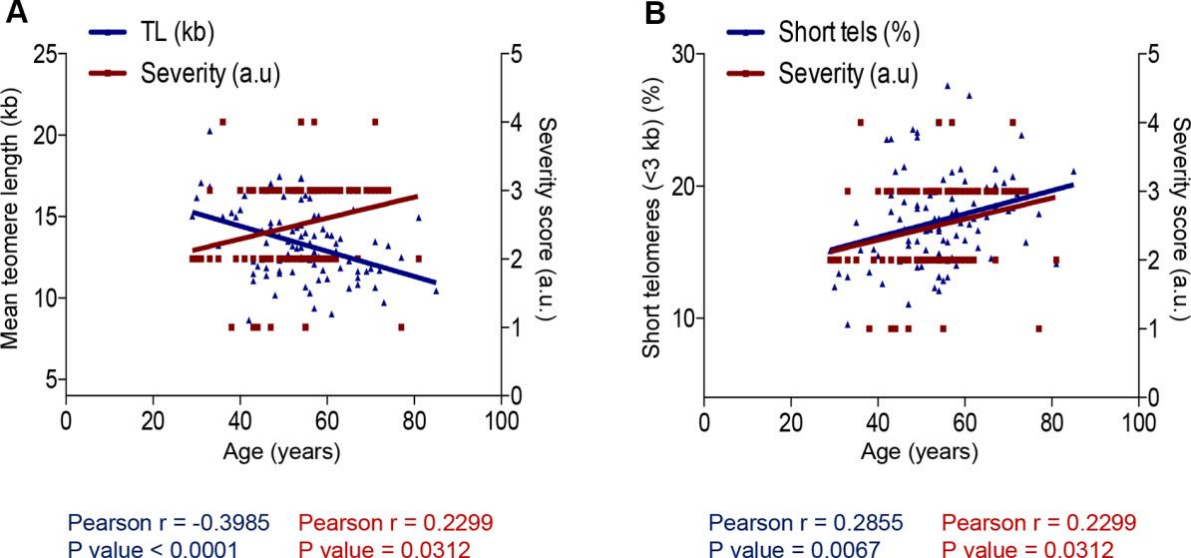
**Correlation between age and COVID-19 severity and telomere length.** (**A**, **B**) Person correlation analysis between age and telomere length measured by HT Q-FISH in PMBC samples (**A**) and with percentage of short telomeres (<3 kb). The severity score was established by assigning values of 1, 2, 3, 4 for mild, moderate, severe and acute, respectively (see Materials and Methods). The Pearson r coefficient and the P values are indicated.

Finally, we also observed a significant direct correlation between age of the COVID-19 patients and the severity score of the disease (r=0.2299; p=0.0312; Figure 4A, 4B). Furthermore, we observed an inverse correlation between age and mean telomere length (TL) and a direct correlation between age and the percentage of short telomeres when using HT Q-FISH (Figure 4A, 4B).

Together, these findings suggest significant correlations of age as well as telomere length with COVID-19 severity.

### Higher severity of COVID-19 disease in patients in the lower percentiles of telomere length

**Figure 5:**
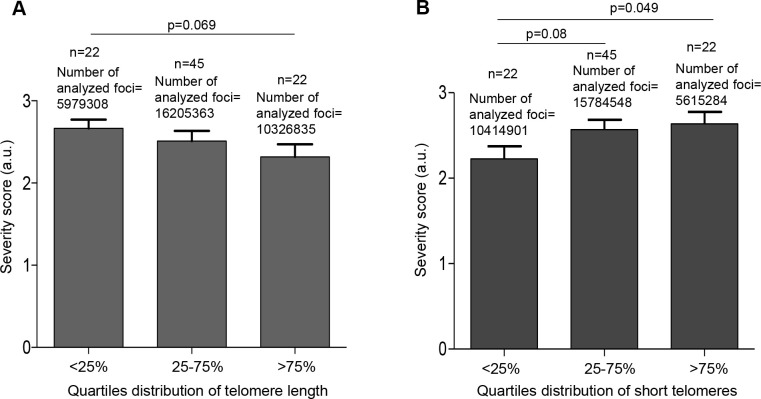
**Patients with shorter telomeres develop more severe COVID-19 disease.** (**A**) The telomere lengths of patients were distributed into the quartiles <25% (<11.68 kb), 25-75% (11.68–14.96 kb) and >75% (>14.96 kb) and correlated with COVID-19 severity. (**B**) The abundance of short telomeres was distributed into the quartiles <25% (<14.73%), 25-75% (14.73-19.32%) and >75% (>19.32%) and correlated with COVID-19 severity. Data represent mean values ±SEM. Statistical significance was assessed using Student’s t test.

The findings suggest that COVID-19 patients with shorter telomere length may have a higher risk of more severe pathologies. To further test this, we divided the patients in quartiles according to either their mean telomere length or their percentage of short telomeres using the telomere signal fluorescence data obtained by HT Q-FISH. We observed that patients in the lower quartile of mean telomere length (<25%) had a higher severity score (p=0.06; Figure 5A). Similarly, the patients in the higher quartile of percentage of short telomeres had significantly higher severity scores of the disease (p=0.049; Figure 5B).

### Different rates of telomere shortening in patients with different severity scores

**Figure 6:**
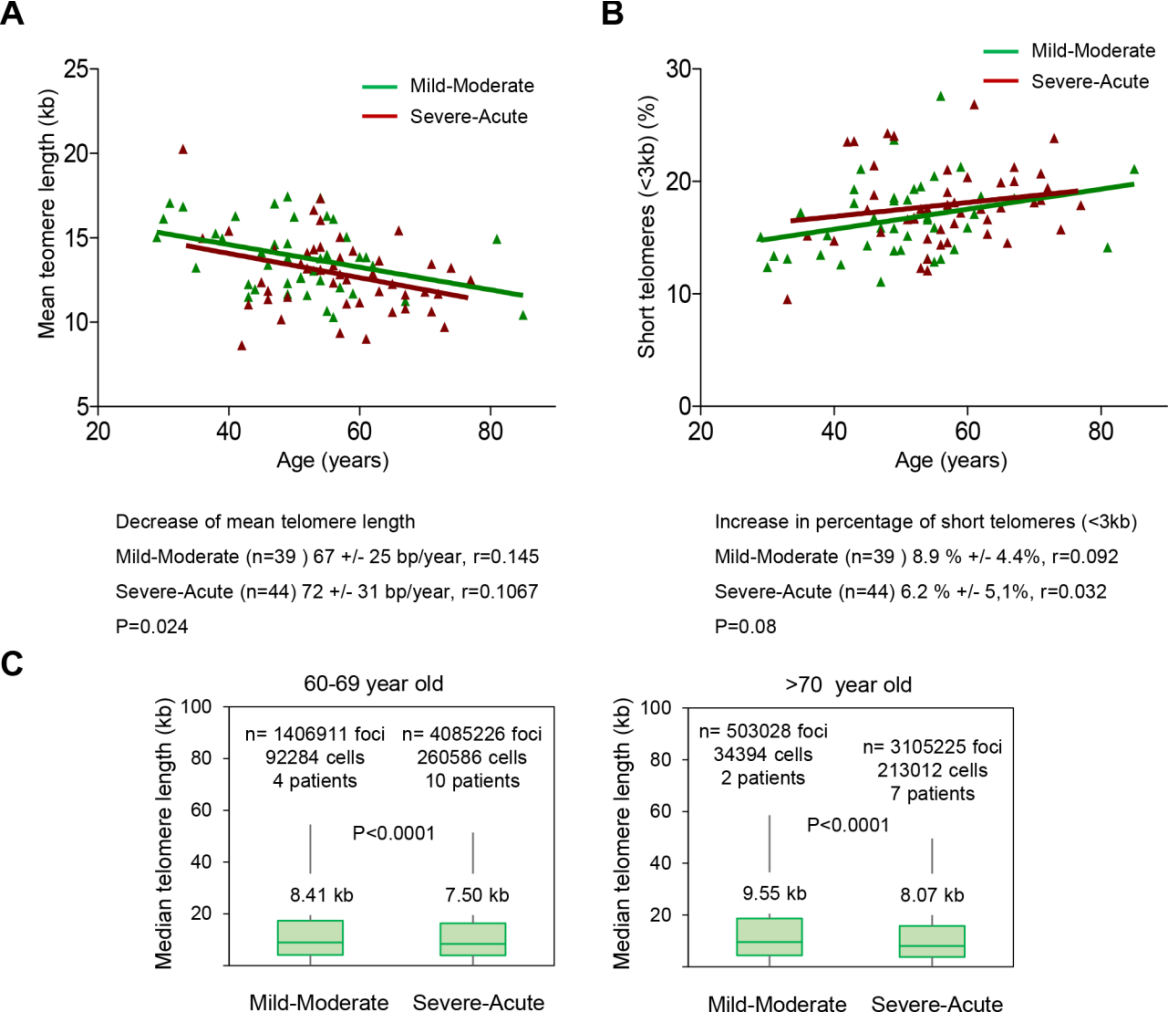
**Patients with a higher COVID-19 severity score show faster telomere shortening rates.** (**A**, **B**) Telomere shortening (**A**) and increase in percent of short telomeres (<3kb) (**B**) with age in patients diagnosed with mild-moderate and severe-acute COVID-19. Linear regression analysis was used to assess the number of bp loss and of the percent of short telomeres per year (<3 kb) in PMBC of these donors. Statistical significance was assessed using the Mann-Whitney test. (**C**) Whisker plot representation of telomere length. The between 60-69 and older than 70-year-old were pooled together within the same age group. The patients diagnosed with mild or moderate and those diagnosed with severe or acute were grouped. The telomere length corresponding to individual telomere foci were plotted according to Covid-19 severity groups. The ends of the box are the upper and lower quartiles so that the box spans the interquartile range. The middle line represents the median and bars to standard deviation. The statistical significance was calculated by one way Anova with post tukey test. n= number of foci.

As patients in the lower quartile of telomere length have a significantly higher risk of severe COVID-19 pathologies, we set to investigate whether the rates of telomere shortening in higher severity score patients were significantly higher than in the lower severity score patients. To this end, we pooled together the patients in “mild-moderate” and “severe-acute” severity groups. We found that patients with a “severe-acute” diagnosis showed a significantly faster rate of telomere shortening compared to the “mild-moderate” diagnosis as determined by HT Q-FISH (p=0.024; Figure 6A). Of note, patients with “severe-acute” COVID-19 disease have shorter telomeres along all age groups compared to patients with “mild-moderate” COVID-19 disease. Similarly, we found an increased rate of accumulation of short telomeres in patients with a “severe-acute” diagnosis compared to patients with a “mild-moderate” severity score (p=0.08; Figure 6B).

In order to address whether this association between Covid-19 severity and short telomeres was independent of the age, we grouped the patients in different age groups (below 40 years of age; between 40-49; 50-59; 60-69 and over 70 years of age) and compared the fluorescence of individual telomere foci in patients showing either “mild-moderate” or “severe-acute” COVID-19 severity. We found that for age groups above 60 years of age, telomeres were shorter in the groups with “severe-acute” severity compared to “mild-moderate” severity (Figure 6C).

## DISCUSSION

Data from COVID-19 around the world shows that patients of older age groups show a higher severity of the disease and a higher mortality. Male patients also show a higher mortality than female patients (see COVID-19 Sex-Disaggregated Data Tracker available at: http://globalhealth5050.org/covid19). This suggest that molecular mechanisms of aging maybe aggravating the pathological consequences of infection by the SARS-CoV-2 virus. Telomere shortening and accumulation of DNA damage steaming from short telomeres has been proposed as one of the primary hallmarks of aging [27]. In particular, short telomeres are known to result in chromosomal instability and loss of cell viability by inducing replicative senescence and/or apoptosis [26]. Importantly, by using mouse models that lack telomerase activity, we and others have shown that short telomeres impair the regenerative capacity of tissues leading to loss of tissue homeostasis and degenerative diseases [40]. Similarly, humans with critically short telomeres owing to mutations in telomerase also show an impaired regeneration capacity and are at a higher risk of developing degenerative diseases in both low proliferative (lung, kidney) and high proliferative tissues (bone marrow, skin) [42].

Given that the SARS-CoV-2 virus infects different cell types in the organisms, including regenerative cell types such as alveolar type II (ATII) cells in the lungs [8–13, 49], here we hypothesize that individuals with short telomeres would have an impaired regenerative response upon SARS-CoV-2 infection, thus leading to more severe and progressive pathologies, such as fibrosis-like pathologies in the lungs, kidney or liver.

To address this, we have measured telomere length in a total of 89 patients diagnosed with COVID-19 ranging from mild to acute disease. As expected we found that telomere length decreases with age, with women having longer telomeres than men at different age groups, which could explain why COVID-19 disease is more severe in males than females. Interestingly, we also found that those patients that have more severe COVID-19 pathologies have shorter telomeres at different ages compared to the patients with milder disease. Indeed, patients which are in the lower percentile of telomere length also have significantly higher severity scores.

These findings demonstrate that molecular hallmarks of aging, such as presence of short telomeres can influence the severity of COVID-19 pathologies. As short telomeres can be elongated by telomerase, and telomerase activation strategies have been shown by us to delay aging and age related pathologies [50], as well as to have therapeutic effects in diseases associated to short telomeres, such as pulmonary fibrosis [44], it is tempting to speculate that such telomerase activation therapies could ameliorate some of the tissue pathologies remaining in COVID-19 patients, such as fibrosis-like pathologies in the lungs [51] after overcoming the viral infection.

## MATERIALS AND METHODS

### Patients

In this study participated a total of 89 patients (61 female and 28 male patients of ages ranging from 29 to 85 years old) from the IFEMA field hospital installed due to the emergency situation in Madrid, Spain. All these samples were donated to CNIO BioBank which allows their use for biomedical analyses under the existing law requirements in Spain.

### Blood samples

A total of 8 ml of blood were collected from the arm of each patient in heparin tubes and 4 ml in EDTA tubes and shipped within less than 24h to the DNA National Bank at Salamanca University, where they were immediately processed at a biosafety level (BSL) 3 (BSL-3) biocontainment level. Peripheral blood mononuclear cells (PBMCs) were purified by Ficoll gradient and frozen in 90% FBS (v/v) supplemented with 10% (v/v) DMSO into a number of aliquots ranging from 1 to 3, according to cell number. PBMCs were stored in vapor phase-nitrogen.

Genomic DNA was extracted directly from blood samples using the Real Blood DNA Kit and stored long-term in TE at -20° C.

### Q-PCR Assay to measure average telomere length

Telomere length was measured in genomic DNA isolated from blood samples. We used a modified monochrome multiplex quantitative polymerase chain reaction (PCR) method already described [45]. Briefly, each reaction included SYBR Green I (Promega), telomere primer pair telg (5´-ACACTAAGGTTTGGGTTTGGGTTTGGGTTTGGGTTAGTGT-3´) and telc (5´-TGTTAGGTATCCCTATCCCTATCCCTATCCCTATCCCTAACA-3´) (final concentrations 900nM each), albumin primer pair albu (5´-CGGCGGCGGGCGGCGCGGGCTGGGCGGaaatgctgcacagaatccttg-3´) and albd (5´-GCCCGGCCCGCCGCGCCCGTCCCGCCGgaaaagcatggtcgcctgtt-3´) (final concentrations 900nM each) and 20 ng of genomic DNA. The Applied Biosystems QuantStudio 6 Flex Real-Time PCR System was used. The thermal cycling profile was Stage 1: 15 min at 95° C; Stage 2: 2 cycles of 15 s at 94° C, 15 s at 49° C; and Stage 3: 32 cycles of 15 s at 94° C, 10 s at 62° C, 15 s at 74° C with signal acquisition, 10 s at 84° C, 15 s at 88° C with signal acquisition. To serve as a reference for standard curve calculation, HeLa cells were serially diluted and qPCR performed as described above. After thermal cycling was completed, the QuantStudio 6 software was used to generate standard curves and Ct values for telomere signals and reference gene signals. The average telomere length was termed T/S ratio. Finally, T/S ratios were converted into kb by external calibration with the K562 (6.5 kb), CCRF-CEM (7.5 kb), Jurkat (11.5 kb) and HeLa1211 (24 kb) cell lines.

### Terminal restriction fragment analysis

Mean telomere length by Telomere Restriction Fragment (TRF) was determined using the method already described [38]. Briefly, genomic DNA was digested by MboI and separated by gel electrophoresis in 0.5X TBE maintained at 14° C, using a CHEF DR-II pulsed-field apparatus (BioRad) for 14 h at 5 V/cm at a constant pulse time of 0.5 s. The gel was transferred to a nylon membrane (Hybond-XL, GE Healthcare) and probed with a ^32^P-labeled telomeric probe (TTAGGG)n (a gift from T. de Lange). Mean TRF lengths were determined using an ImageQuant TL.

### HT Q-FISH

Clear bottom black-walled 96-well plates (Greiner, Longwood, FL) were precoated with a 0.001% (wt/vol) poly-L-lysine solution (Sigma-Aldrich, St. Louis, MO) for 1h at 37° C. Poly-L-lysine was removed and wells rinsed with RPMI before cell addition (75,000–150000 lymphocytes/well).

PBMCs were thawed in complete RPMI 1640 growth media supplemented with 10% FBS (v/v) and seeded at a concentration of 100000 cells/ well in triplicate wells per sample. Cells were left to adhere to the plate for 1 h at 37C in incubator with 95% humidity, 5% CO_2_. Plates were then removed from incubator and then fixed at room temperature (RT) by slowly filling up the wells with 200 ul methanol/acetic acid (3/1, vol/vol) and incubated for 10 to 15 min. The solution was removed, and this was repeated 2 more times, leaving the last fixative volume up for a total of 1 h fixation. Plates were then moved to -20 until processed for high-throughput quantitative FISH (HT Q-FISH).

We performed HT Q-FISH as described before [46]. Briefly, the plates were removed from -20, the fixative solution removed, and the plates were dried on a hot plate at 37° C overnight, followed by rehydration with 200 μL of PBS. Cells were fixed with 200 μL of 4% formaldehyde in PBS for 2 min at room temperature (RT) and washed 3 times for 5 min with PBS. Prewarmed pepsin solution (100 mL of H_2_O, 100 μL of 37% HCl [10.1 M HCl], and 100 mg of pepsin [Sigma-Aldrich; catalog no. P7000-25G]) was used to degrade cell walls for 15 min at 37° C followed by 2 washing steps of 5 min with 200 μL of PBS. The cells were then dehydrated with sequential 5-min 70%, 90%, and 100% ethanol steps. The plates were dried 1 h at 37° C. Next, 50 μL of the hybridization solution containing the Tel-Cy3 PNA probe was added to the plates (95 μL of 1 M Tris, pH 7.0, 812 μL of MgCl_2_ solution [25 mM MgCl_2_, 9 mM citric acid, 82 mM Na_2_HPO_4_], 6.65 mL of deionized formamide, 475 μL of blocking reagent [10 g of blocking reagent (Boehringer; catalog no. 1093 657) dissolved with heat in 100 mL of maleic acid buffer, pH 7.5 (100 mM maleic acid, 150 mM NaCl)], 1.28 mL of H_2_O, and 190 μL of Tel-Cy3 PNA probe [5 μg lyophilized Cy3-(C3TA2)3 PNA probe (Panagene) diluted in 200 μL of H_2_O]). Plates were then sealed with aluminum foil lids. The DNA was denatured by heating the plate on a hot plate at 85° C for 5 min and left to incubate for 2 h at RT in the dark. The plates were rinsed and washed in plate shaker with wash solution 1 (10 mM Tris-HCl pH 7, 70% Formamide, 0.10% BSA in H_2_O) for 30 min, followed by 3 washes of 5 min each with wash solution 2 (TBS [Tris-buffered saline, pH 7.0] with 0.08% Tween 20). Nuclei staining was performed incubating for 10 min with TBST containing 1 μg/mL DAPI (4′,6-diamidino-2-phenylindole, dihydrochloride; Life Technologies; catalog no. D-1306). Then the plates were washed 1 × 5 min with PBS and stored at 4° C in the dark. Images from the plate were then acquired by HT microscopy within 48 h.

### High-throughput microscopy

Quantitative image acquisition was performed on an Opera High Content Screening System (PerkinElmer) 40×/0.9 N.A. water-immersion objective. UV and 561 nm excitation wavelengths were used to detect DAPI and Cy3 telomeric signals, respectively and 60 independent images were captured at different positions of each well. Images were analyzed with Acapella Image analysis software (PerkinElmer). Data were analyzed with SPSS (IBM) and Excel (Microsoft). Telomere fluorescence values were converted into kilobases by external calibration with the CCRF-CEM (7.5 kb), L5178Y-S (10.2 kb), L5178Y-R (79.7 kb) and Jurkat (11.5 kb) cell lines.

### Criteria for the diagnosis of COVID-19

Depending on the clinical features of COVID-19, patients are generally divided as mild, moderate, severe and acute.

1. Mild COVID-19: low-grade fever, cough, malaise, rhinorrhea, sore throat with or without hemoptysis, nausea, vomiting, diarrhea, but without any radiological features of pneumonia and absence of mental changes.

2. Moderate COVID-19: fever, respiratory symptoms including dry cough and shortness of breath that may emerge along with the radiological features.

3. Severe COVID-19: dyspnea, respiratory frequency ≥30/minute, blood oxygen saturation ≤93%, PaO2/FiO2 ratio <300, and/or lung infiltrates >50% of the lung field within 24–48 h.

4. Acute COVID-19: usually develops after 7 days in patients with mild/moderate/severe COVID-19 with features of Acute respiratory distress syndrome (ARDS) requiring mechanical ventilation along with presence of multiorgan dysfunction failure, metabolic acidosis and coagulation dysfunction.

### Data availability statement

The data that support the findings of this study are available from the corresponding author upon reasonable request.
